# Network connections, dyadic bonds and fitness in wild female baboons

**DOI:** 10.1098/rsos.160255

**Published:** 2016-07-27

**Authors:** Dorothy L. Cheney, Joan B. Silk, Robert M. Seyfarth

**Affiliations:** 1 Department of Biology, University of Pennsylvania, Philadelphia, PA 19104, USA; 2 Department of Psychology, University of Pennsylvania, Philadelphia, PA 19104, USA; 3 School of Human Evolution and Social Change, Arizona State University, Tempe, AZ 85287, USA

**Keywords:** baboons, females, fitness, dyadic bonds, social connections

## Abstract

In many social mammals, females who form close, differentiated bonds with others experience greater offspring survival and longevity. We still know little, however, about how females' relationships are structured within the social group, or whether connections beyond the level of the dyad have any adaptive value. Here, we apply social network analysis to wild baboons in order to evaluate the comparative benefits of dyadic bonds against several network measures. Results suggest that females with strong dyadic bonds also showed high eigenvector centrality, a measure of the extent to which an individual's partners are connected to others in the network. Eigenvector centrality was a better predictor of offspring survival than dyadic bond strength. Previous results have shown that female baboons derive significant fitness benefits from forming close, stable bonds with several other females. Results presented here suggest that these benefits may be further augmented if a female's social partners are themselves well connected to others within the group rather than being restricted to a smaller clique.

## 1. Introduction

A predominant hypothesis in studies of animal social cognition is that natural selection has favoured individuals who have the skill and motivation to compete effectively with others, to monitor others' relationships and to recognize others' motivations and intentions [1–4]. These abilities are thought to facilitate the formation of adaptive social bonds. Indeed, there is accumulating evidence in many mammalian species that the establishment and maintenance of close social bonds varies among individuals, and that these social bonds enhance reproductive success and longevity (baboons [5], house mice [6], horses [7], Assamese macaques [8], dolphins [9], hyaenas [10], reviewed by Seyfarth & Cheney [11]). In our long-term study of free-ranging baboons, for example, we used information about the rate of several affiliative behaviours to construct a composite sociality index (CSI) that measured the strength of bonds between pairs of females [12]. We found that females who had the strongest ties to other females on average experienced greater offspring survival, while those who maintained the strongest and most enduring ties to their top partners experienced greater longevity [13]. The variation among females in bond strength could not be explained solely by dominance rank or the presence of kin.

We still have a poor understanding, however, about the factors—cognitive or otherwise—that might contribute to variation in bond formation. In particular, we are only beginning to consider whether knowledge of others' relationships helps animals to form strategic social connections, including, in particular, links that extend beyond the level of the dyad.

While dyadic analyses are valuable in assessing the strength of pair-wise relationships, they provide little information about how females' social networks are structured. A female's interactions might be restricted to partners who are all members of the same small clique. Alternatively, each of her partners might associate with an entirely different set of individuals. In the former case, a female's interactions would show a high degree of clustering. In the latter, they would demonstrate greater connectivity within the group as a whole. Similar questions have arisen in individual-based analyses that focus on overall levels of social behaviour rather than on their distributions. In one study of yellow baboons, for example, females who interacted with other adults at high frequencies lived longer than less social females [14]. Whether high levels of sociality resulted from strong bonds with a few individuals or weaker bonds with many, however, was not clear.

Although numerous studies have attempted to examine the adaptive consequences of connections beyond the level of the dyad [4], there is still no consensus on which measures in social network analyses (SNAs) are most meaningful [15–21]. Several studies of species living in fission–fusion societies have shown that individuals who connect otherwise unconnected individuals (i.e. have high levels of ‘betweenness’) experience greater mating success (e.g. long-tailed manakins [22], chimpanzees [23]). These individuals are hypothesized to be influential ‘power brokers’ within their communities. Other studies have shown that the individuals who enjoy greater fitness are those who display high levels of ‘clustering’ (or ‘transitivity’), by forming associations with their partners' associates (e.g. hyaenas [10], rock hyrax [24,25], humans [26,27]). By contrast, among male juvenile dolphins the strongest predictors of survival were an individual's connections to other well-connected individuals (eigenvector centrality) [28].

Social connections beyond the level of the dyad might also be important in species that live and travel as a cohesive unit, including Old World monkeys. In semi-free-ranging rhesus macaques, mothers with higher eigenvector centrality (as measured by proximity scores) tended to experience higher infant survival [4,29]. Conversely, individuals with weak eigenvector centrality often carried a genetic variant associated with low serotonergic signalling.

In short, although the data-linking social network measures with adaptive outcomes remain limited, it seems likely that optimum patterns of social connections vary across different social structures. To date, however, no study has compared the explanatory value of analyses based solely on dyadic interactions with those based on SNA. It remains possible that analyses that consider connections beyond the level of the dyad provide little added value to the information already revealed by dyadic analyses—at least for those species that live in cohesive social groups.

Here, we apply SNA to long-term data from a group of free-ranging baboons, in an exploratory attempt to address three questions. First, can SNA tell us anything that has not previously been revealed by analyses based on measures of dyadic interactions, like the CSI? Second, which network measures are the most informative? Finally, which, if any, network measures are correlated with measures of fitness? To address these questions, we first examine the relationship between females' CSI scores and their scores on four network measures that consider connections beyond the level of the dyad. We then examine the explanatory power of these network measures, using offspring survival as one measure of reproductive success.

## 2. Material and methods

Data were derived from a long-term study of wild chacma baboons (*Papio hamadryas ursinus*) in the Moremi Game Reserve, Botswana [2]. The group had been observed since 1978 and was studied intensively from 1992 to 2007. The number of adult females (aged more than 5 years) in the group ranged from 19 to 26. Maternal kinship was known for all individuals. The primary causes of mortality were infanticide and predation [30]. Predation was estimated to account for more than 95% of all deaths among adults and juveniles. There was no evidence that climatic factors affected females' mortality rates or stress levels (as measured by faecal glucocorticoids; [31]).

As in other cercopithecine primates, female baboons are philopatric and assume dominance ranks similar to their mothers' [2,32]. Dominance ranks remain relatively stable across years. Groups move as a unit, and individuals come into daily proximity with all other group members. However, social relationships are highly differentiated, and females form close bonds with only a few other individuals [12,33].

Behavioural and network analyses were based on 7 years for which we had continuous focal animal sampling (2001–2007) of 49 adult females, yielding a total of 192 female years. During this period, annual sociality index scores (CSI) for each female dyad were constructed as described in Silk *et al.* [12,33,34]. The CSI (or DSI; [34]) is a composite index based on the rate of approaches, groom presents, grooming initiations and the duration of grooming given and received within dyads. In network analyses, dyadic CSI scores in each year were used as weighted edges connecting nodes (females) with other females. From these weighted edges, we calculated four network measures (see below) for each female in each year. We then compared each female's average dyadic CSI scores with all other females in a given year with her network scores for that year (see the electronic supplementary material, Methods). In analyses that considered dominance ranks, we used females' average ranks across years.

For each female in each year, we calculated four network measures that describe social connections beyond the level of the dyad [4]:

*Betweenness*: measures the total number of shortest paths that pass through an individual linking other group members with each other. Individuals with high betweenness connect otherwise unconnected animals. In some societies, betweenness may be important for the transmission of information or disease [21,35,36].

*Eigenvector centrality*: measures the extent to which an individual's partners are connected to others in the network. Each node's centrality is the sum of the centrality values of the nodes to which it is connected. Individuals with high eigenvector centrality have connections with partners who themselves have a large number of partners. (http://demonstrations.wolfram.com/NetworkCentralityUsingEigenvectors/.)

*Clustering coefficient* (*transitivity*): measures the proportion of a subject's partners who are partners with each other. Individuals with high clustering coefficients are more cliquish.

*Reach*: measures the number of other individuals an individual can reach in *k* or fewer steps (degrees of separation). Individuals with high reach are connected to many others who are *k* steps away.

We used linear mixed models (LMMs) with a Gaussian error structure so that we could directly compare results obtained from network analysis with those obtained previously from our analysis based on dyads (CSI) [12]. We used these models to assess the relative importance of the four social network parameters and two possible covariates (dominance rank and the presence of kin), in accounting for variation in the CSI (electronic supplementary material, Methods). Female identity and year were entered as random factors, with appropriate corrections for both intercept and slope [37]. All measures were *z*-scored within a given year for statistical analyses. Thus, each female's annual *z*-score on a particular measure reflected her score relative to other females in that year. None of the network measures were significantly correlated with each other (electronic supplementary material, table S1).

To compare the relative importance of network measures and the CSI in accounting for variation in offspring survival, we used data derived from the Cox proportional hazards model analysis previously presented in Silk *et al.* [12]. In this analysis, offspring were the unit of analysis and survival was calculated through 10+ years (*n* = 148 offspring; censored offspring = 91; uncensored offspring = 57; *n* = 41 mothers; electronic supplementary material). Because some females contributed more than one offspring to the dataset, we used the cluster option to control for the effects of maternal identity [12] (electronic supplementary material, Methods). Global tests of the proportional hazards assumption based on Schoenfeld residuals [38] were not significant (*χ*^2^ = 0.04, d.f. = 1, *p* > 0.843), indicating that the analysis did not violate model assumptions.

The original Cox proportional hazards analysis as described in Silk *et al.* [12] was conducted in STATA (11.0; Statcorp 2009). Statistical analyses for the current study were conducted using R package lmerTest for LMM and the anova function for model comparison. Network measures were calculated using R package igraph (R v. 3.12, R Foundation for Statistical Computing, R Development Core Team, 2009).

For comparison, in table 3 of the main text and electronic supplementary material, table S2, we present the results of AIC model testing on many of the same data [39].

## 3. Results

### 3.1. Network measures and composite sociality index

**Figure 1.**
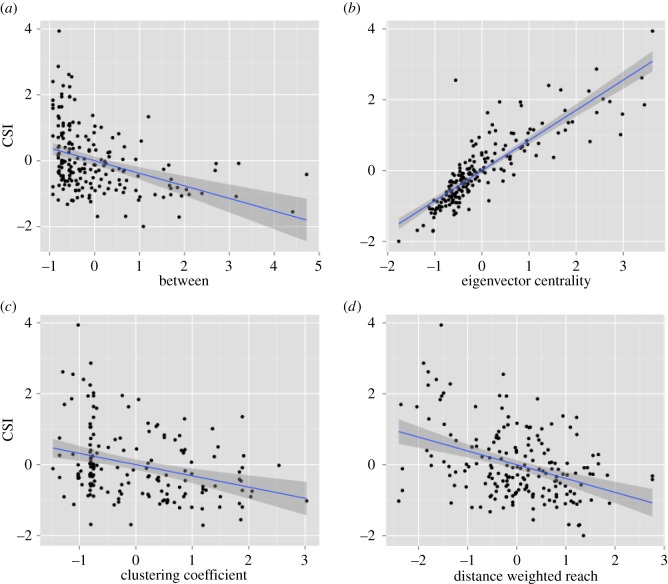
Scatter plots depicting the correlation between the CSI and (*a*) betweenness, (*b*) eigenvector centrality, (*c*) clustering coefficient and (*d*) reach. Shaded areas show standard errors of the smoothed curve.

Figure 1 illustrates the relationships between network measures and CSI, and table 1 presents results of an LMM using all network measures as predictors and CSI as the dependent measure. We used a likelihood ratio test to compare this full model with a null model that included only the random effects [40]. The full model fit the data better than the null model (*χ*^2^ = 98.89, d.f. = 4, *p* < 0.01). In the full model, eigenvector centrality was significantly positively correlated with CSI and reach was significantly negatively correlated with CSI. There was also a negative, but non-significant, relationship between CSI and both clustering coefficient and betweenness (figure 1 and table 1). Thus, females who were connected to other highly connected individuals had significantly higher CSI scores than others. Similar results emerged from AIC-based model comparisons (electronic supplementary material, table S2).

**Table 1.** Results of a multiple regression in which the four network measures served as predictor values and CSI was the dependent variable. Scores were calculated annually for each measure. *N* = 192 female years, including 49 unique females who were observed from between 1 and 7 years.

	estimate (*β*)	s.e.	*t*-value	*p*-value
betweenness	−0.079	0.048	−1.643	0.104
eigenvector centrality	0.779	0.058	24.020	<0.0001
clustering coefficient	−0.113	0.056	−2.033	0.055
reach	−0.154	0.059	−2.622	0.013

Females' eigenvector centrality scores tended to be consistent across years. There were 143 cases in which a female was observed in two successive years; the correlation between a female's score from one year to the next was significantly positive (*β* = 0.275, s.e. = 0.107, *t* = 2.558, *p* = 0.015). No other network measures displayed this significant level of within-subject consistency.

There was no relationship between any of the network measures and female dominance rank. However, females with close kin (mothers, adult daughters, adult sisters) present in the group were significantly more likely to have a high eigenvector centrality (*β* = 0.291, s.e. = 0.104, *t* = 2.811, *p* = 0.006). This finding is not surprising, given previous results indicating that CSI scores were positively correlated with the presence of kin, but not with dominance rank [12]. By contrast, the presence of kin was negatively correlated with clustering coefficient (*β* = −0.194, s.e. = 0.099, *t* = −1.958, *p* = 0.054). There was no relationship between the presence of kin and reach or betweenness.

### 3.2. Network measures and offspring survival

Table 2 presents results of an LMM using all network measures as predictors and offspring survival (calculated from the Cox proportional hazard model [12]) as the dependent measure. Eigenvector centrality was the only measure that was significantly correlated with offspring survival.

**Table 2.** Results of a multiple regression in which the four network measures served as predictors and offspring survival was the dependent variable. Negative coefficients represent variables that are associated with reduced mortality. *N* = 148 offspring, born to 41 mothers.

	estimate (*β*)	s.e.	*z*-value	*p*-value
between	−0.377	0.215	−1.75	0.079
eigenvector centrality	−0.347	0.108	−3.20	0.001
clustering coefficient	0.186	0.182	1.02	0.307
reach	0.126	0.259	0.49	0.626

### 3.3. Network measures compared with composite sociality index

The high positive correlation between CSI (a dyadic measure) and eigenvector centrality (a network measure) raised the possibility that the primary determinant of offspring survival in our analysis was a female's CSI, and that her partners' connections were relatively unimportant. Thus, we wanted to examine the source of the variation in offspring survival among individuals: did it arise from the strength of females' dyadic bonds only (high CSI), or was there any added value to their secondary bonds (high eigenvector centrality)?

To address this question, we used AIC-based model testing to compare the relative importance of network measures and CSI in predicting offspring survival. In this analysis (table 3), the best model did not include CSI but instead included eigenvector centrality and betweenness as predictors. Although there was little difference in predictive power among the five best models, eigenvector centrality was included in all five models, whereas CSI appeared only in the fifth best model (table 3). Thus, network measures, particularly eigenvector centrality, appeared to account for variation in offspring survival above and beyond that accounted for by CSI alone (see the electronic supplementary material, table S3 for a complementary analysis examining the comparative effects of CSI and residual values of eigenvector centrality scores on infant survival).

**Table 3.** Results of model testing using all possible combinations of predictors (*N* = 31) and offspring survival (calculated using the Cox proportional hazard model) as the dependent measure. Results for the best five models are shown. *N* = 148 offspring, born to 41 mothers. ∑ *W*_*I*_ provides the summed values of the model weights for all of the models in which each variable was included.

model rank	betweenness	eigenvector centrality	clustering coefficient	reach	CSI	AIC	Δ_i_	model weight (*w*_*i*_)
1	X	X				705.9	0	0.113
2		X				706.3	0.403	0.093
3	X	X	X			707.0	1.130	0.064
4		X	X			707.1	1.273	0.060
5		X	X		X	707.3	1.444	0.055
∑ *W*_*I*_	0.49	0.73	0.30	0.41	0.44			

## 4. Discussion

The primary aim of this analysis was to examine whether network measures of females' social bonds could reveal any new information about their sociality and reproductive success that was not already demonstrated by dyadic measures. The answer seems to be a qualified ‘yes’: females who formed bonds with individuals who were themselves well connected within the group had higher CSI scores and experienced greater offspring longevity. A high eigenvector centrality score seemed to provide added value to the fitness benefit deriving from close dyadic bonds. By contrast, females with high clustering coefficients had low CSI scores, suggesting that females with weaker social bonds were apt to form relationships within the same small subgroups. CSI was also negatively correlated with betweenness and reach; thus, simply being connected to many other females was not necessarily associated with stronger social bonds.

Our results suggest some potentially important differences in adaptive patterns of social bonds in species that live in fission–fusion societies (e.g. hyaenas, chimpanzees) as opposed to cohesive social groups (e.g. baboons, macaques). Hyaenas tend to exhibit high clustering, associating with ‘friends’ who are also friends with each other ([10]; see also [25] for similar data on rock hyraxes). Such clustering may facilitate cooperation in communities where group members are often widely dispersed and do not encounter each other on a daily basis. The ability to serve as a link between otherwise unconnected individuals (high betweenness) may also be of high functional value in fission–fusion societies, as has been suggested for chimpanzees ([23], see also [35,36,41]). By contrast, female baboons come into predictable contact with one another on a daily basis. As a result, the most successful individuals may be those who are well connected with partners who themselves are well connected, as opposed to those who are relatively isolated within a small clique [4,29]. It remains for future research to investigate these questions in more detail.

The proximate mechanisms underlying the benefits of secondary connections compared to the benefits of strong dyadic bonds remain unclear. It seems possible that having preferred partners who are themselves the preferred partners of several others may facilitate social interactions by making encounters more predictable and less stressful. Similarly, such connections may facilitate the formation of new bonds if a female's primary partner dies. The offspring of individuals with high eigenvector centrality may also be less peripheral and at lower risk of predation—the primary cause of mortality for juveniles and adults in this population [30].

In sum, we evaluated the comparative benefits of dyadic bonds and bonds beyond the level of the dyad by comparing our previous results with new data using network analysis. Whereas our previous results showed that female baboons derive significant fitness benefits from forming close, stable bonds with several other females, network analysis indicates that these benefits may be augmented if a female's social partners are themselves well connected to many others within their group, rather than being clustered in small cliques. Whether variation in the ability to monitor other individuals' relationships contributes to variation in the ability to form connections with influential partners remains to be explored.

## Supplementary Material

SI: Methods and Tables

## Data accessibility

The dataset supporting this article is available at https://sites.sas.upenn.edu/seyfarth/.
