# Supplementary material for: Network connections, dyadic bonds and fitness in wild female baboons
Source: R Soc Open Sci. 2016 Jul 27;3(7):160255. doi: 10.1098/rsos.160255 (PMC4968471; doi:10.1098/rsos.160255)
Supplement: SI: Methods and Tables [file rsos160255supp1.docx]

Supplementary information (SI)

Methods

Methods for calculating CSI were the same as those described in Silk et al. (2009), with two exceptions. First, to control for possible annual variation in interaction rates, we normalized (z-scored) annual CSI and network measures. Second, because females’ average annual network scores were derived from their interactions with all other females in the group, females’ average annual CSI scores were calculated using all possible partners rather than only their top 3 partners (the dependent variable used in Silk et al. 2009). Because females’ relationships were highly differentiated (Silk et al. 2009, 2010a), these two measures were highly positively correlated)(β = 0.867, se: 0.039, t = 22.05, P < 0.001).

We included all infants in the sample of offspring survival, including those who died before 100 days of age. The sample of 148 offspring included 57 censored individuals (31 individuals aged >5 years, 15 individuals aged 1-5 years, and 11 individuals <1 year). Because adolescent males who disappeared may have dispersed rather than died, males who disappeared after 8 years of age were censored (N = 8). When comparing offspring longevity and CSI, we calculated a female’s average CSI score across years (Silk et al. 2009).

Model testing

We also used model-testing procedures to assess the relative importance of the four social network parameters in accounting for variation in the sociality index (CSI), and the relative importance of network parameters and CSI in accounting for infant survival. We evaluated models that included all possible combinations of the four or five normalized predictor variables, and calculated the AIC values for each model. We identified the model with the lowest AIC score, and subtracted the minimum AIC score from the AIC score for each of the other models to obtain ΔAIC values.

We computed the weight of each model using the formula:


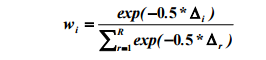


where Δ_i_  is the ΔAIC value for a model *i.* Then, we summed the model weights for all models that included each variable to obtain an estimate of a probability that a given variable was included in the best model (Wagenmakers & Farrell 2004).

Table 1. The correlation among fixed effects.

|  | Betweenness | Eigenvector centrality | Clustering  coefficient |
| --- | --- | --- | --- |
| Eigenvector centrality | 0.124 |  |  |
| Clustering coefficient | -0.077 | 0.191 |  |
| Reach | -0.482 | 0.105 | 0.069 |

Table 2. Results of model testing using all combinations of network measures as predictors (N =15) and CSI as the dependent measure. The five best models are shown. The best model used eigenvector centrality, clustering coefficient, and reach as predictors. It was slightly better than the second-best model, which used all four network measures as predictors. There was a moderate decline in predictive power with the third-best model; all other models were much less effective.

| Model  rank | Betweenness | Eigenvector  centrality | Clustering  coefficient | Reach | AIC | Δ_i_ | Model  weight (*w_i_*) |
| --- | --- | --- | --- | --- | --- | --- | --- |
| 1 |  | X | X | X | 252.7 | 0 | 0.628 |
| 2 | X | X | X | X | 254.1 | 1.4 | 0.312 |
| 3 | X | X | X |  | 257.4 | 4.7 | 0.060 |
| 4 |  | X |  | X | 278.8 | 26.1 | <0.001 |
| 5 | X | X |  |  | 279.9 | 27.2 | <0.001 |
| **∑ W*_I_*** | 0.37 | 1.0 | 0.99 | 0.94 |  |  |  |

∑ W*_I_* provides the summed values of the model weights for all of the models in which each variable was included. N = 192 female years, including 49 unique females who were observed from between 1-7 years.

Table 3. Results of a LMM in which females’ CSI and residual eigenvector centrality scores served as predictor variables and offspring survival was the dependent measure. Both predictors were significantly related to infant survival, but the effect of females’ eigenvector centrality residual scores was stronger than that of CSI. Thus, females with higher offspring survival had higher eigenvector centrality scores than would have been predicted from their CSI scores alone.

|  | Estimate | Std. error | z value | P |
| --- | --- | --- | --- | --- |
| CSI | -0.194 | 0.099 | -1.96 | 0.050 |
| Eigenvector centrality residual | -0.508 | 0.201 | -2.52 | 0.012 |

Residual scores were based on the regression between eigenvector centrality scores and CSI values. N = 141 infants, born to 40 mothers.

References

Silk JB, Beehner JC, Bergman TJ, Crockford C, Engh AL, Moscovice LR, Wittig RM, Seyfarth RM, Cheney DL. 2009 The benefits of social capital: close social bonds among female baboons enhance offspring survival. *Proc Roy. Soc. B* **276**, 3099-3104. (doi: 10.1098/rspb.2009.0681)

Silk JB, Beehner JC, Bergman TJ, Crockford C, Engh AL, Moscovice LR ,Wittig RM, Seyfarth RM, Cheney DL. 2010b Strong and consistent social bonds enhance the longevity of female baboons. *Curr. Biol.* **20**, 1359-1361.

Wagenmakers E-J, Farrell S. 2004 AIC model selection using Akaike weights. *Psychonom. Bull. Rev*. **11**, 192-196.
